# Changes in the Pulp Tissue Complex Induced by Orthodontic Forces: Is There a Need for Concern? A Systematic Review and Meta-Analysis of RCTs and Prospective Clinical Trials

**DOI:** 10.3290/j.ohpd.b3556039

**Published:** 2022-11-08

**Authors:** Isabella Scherer, Giorgos N. Tzanetakis, Theodore Eliades, Despina Koletsi

**Affiliations:** a Postgraduate Student, Clinic of Orthodontics and Pediatric Dentistry, Center of Dental Medicine, University of Zurich, Zurich, Switzerland. Design and planning, performed experiment, collected data, wrote the manuscript, read and approved the final manuscript.; b Assistant Professor, Department of Endodontics, School of Dentistry, National and Kapodistrian University of Athens, Athens, Greece. Study conception, design and planning, performed the experiment, wrote the manuscript, read and approved the final manuscript.; c Professor and Director, Clinic of Orthodontics and Pediatric Dentistry, Center of Dental Medicine, University of Zurich, Zurich, Switzerland. Study conception, design and planning, performed experiment, wrote the manuscript, read and approved the final manuscript.; d Senior Teaching and Research Staff, Clinic of Orthodontics and Pediatric Dentistry, Center of Dental Medicine, University of Zurich, Zurich, Switzerland. Study conception, design and planning, performed experiment, collected and analyzed data, wrote the manuscript, read and approved the final manuscript, project administration.

**Keywords:** orthodontic force, meta-analysis, pulp tissue, systematic review

## Abstract

**Purpose:**

To identify and assess any changes in the pulp tissue complex following orthodontic force application.

**Materials and Methods:**

Published and unpublished literature was searched in seven databases until 9 August 2022 for randomised controlled trials (RCTs) and prospective trials (nR-PCT). Representative key words included ‘pulp response’, ‘pulp tissue’, ‘orthodontic force’, and ‘tooth movement’. Study selection, data extraction, risk of bias and certainty of evidence assessment were conducted independently by two reviewers. Random effects meta-analyses with respective confidence intervals (95%CIs) were conducted where applicable.

**Results:**

A total of 363 records were screened, a final number of 24 articles were eligible for qualitative synthesis, while 8 of those contributed to meta-analyses. There was evidence that pulpal blood flow (PBF) decreased after 3 weeks of tooth movement compared to no force application (4 studies, mean difference: -1.68; 95% CI: -3.21, -0.15; p = 0.03). However, this was not the case after 6 months of treatment (p = 0.68). A rise in the activity of aspartate aminotransferase (AST) was detected after 7 days of treatment, but combining 2 studies, this was not statistically significant (p = 0.25). Other outcomes were assessed through single studies. Risk of bias was within the range of ‘some concerns/moderate to high/critical overall’, while certainty of evidence was low to very low according to GRADE.

**Conclusions:**

As a short-term effect, PBF decreased upon initiation of orthodontic force application, while enzymatic and peptide activity within the pulp was transiently affected. Further long-term evidence of improved quality and certainty is needed.

**Supplementary Table 1:** Detailed assessment of RoB 2.0 tool

Domain	Reference	Abu Alhaija et al, 2019	Abu Alhaija et al, 2021	Al-Ainawi et al, 2016	Caviedes-Bucheli et al, 2021	Chavarria et al, 2014	Ersahan and Sabuncuoglu 2015	Han et al, 2013	Han et al, 2020	Hatrom et al, 2021	Mostafa et al, 1991	Ramazanzadeh et al, 2008	Sabuncuoglu and Ersahan 2014a
1. Randomization process	1.1	Y	Y	Y	Y	Y	PY	PY	PY	Y	PY	PY	PY
	1.2	NI	Y	Y	NI	NI	NI	NI	NI	NI	NI	NI	NI
	1.3	PN	N	N	PN	PN	PN	PN	PN	PN	NI	NI	NI
	Assessor’s judgement	Some concerns	Low	Low	Some concerns	Some concerns	Some concerns	Some concerns	Some concerns	Some concerns	Some concerns	Some concerns	Some concerns
2. Deviations from intended interventions	2.1	Y	NI	NI	PY	PY	NI	NI	NI	Y	NI	PY	NI
	2.2	Y	Y	PY	PY	PY	NI	NI	NI	Y	NI	PY	NI
	2.3	PY	N	PN	N	PY	PN	PN	PN	PN	PN	PN	PN
	2.4	PN	-	-	-	PY	-	-	-	-	-	-	-
	2.5	-	-	-	-	PN	-	-	-	-	-	-	-
	2.6	PN	PN	PN	PY	PN	PY	PY	PY	PN	NI	PY	PY
	2.7	PN	N	N	-	NI	-	-	-	PN	NI	-	-
	Assessor’s judgement	Some concerns	Some concerns	Some concerns	Low	High	Low	Low	Low	Some concerns	High	Low	Low
3. Missing outcome data	3.1	N	N	N	Y	PN	Y	PY	PY	PY	NI	Y	Y
	3.2	PN	PN	PN	-	PN	-	-	-	-	NI	-	-
	3.3	PN	PN	PN	-	PY	-	-	-	-	NI	-	-
	3.4	-	-	-	-	NI	-	-	-	-	NI	-	-
	Assessor’s judgement	Some concerns	Some concerns	Some concerns	Low	High	Low	Low	Low	Low	High	Low	Low
4. Measurement of the outcome	4.1	N	N	N	N	PN	PN	PN	PN	PN	PN	PN	N
	4.2	N	N	N	N	NI	NI	NI	NI	N	PN	PN	NI
	4.3	NI	NI	N	N	NI	NI	NI	NI	N	PN	N	NI
	4.4	PY	PY	-	-	NI	NI	NI	NI	-	-	-	NI
	4.5	PN	PN	-	-	PN	PN	PN	PN	-	-	-	PN
	Assessor’s judgement	Some concerns	Some concerns	Low	Low	Some concerns	Some concerns	Some concerns	Some concerns	Low risk	Low	Low	Some concerns
5. Selection of the reported result	5.1	PY	PY	PY	PY	PY	PY	PY	PY	PY	NI	NI	PY
	5.2	NI	PN	PN	PN	NI	NI	NI	NI	PN	NI	NI	NI
	5.3	NI	PN	PN	PN	NI	NI	NI	NI	PN	NI	NI	NI
	Assessor’s judgement	Some concerns	Low	Low	Low	Some concerns	Some concerns	Some concerns	Some concerns	Low risk	Some concerns	Some concerns	Some concerns
Overall	Assessor’s judgement	Some concerns	Some concerns	Some concerns	Some concerns	High	Some concerns	Some concerns	Some concerns	Some concerns	High	Some concerns	Some concerns
Notes		Protocol not registered	Registered Protocol	Registered Protocol	Registered protocol	Protocol not registered	Protocol not registered	Protocol not registered	Protocol not registered	Registered protocol	Protocol not registered	Protocol not registered	Protocol not registered

Y, yes; N, no; PY, probably yes; PN, probably no; NI, no information

**Supplementary Table 2:** Detailed assessment of ROBINS-I tool

Domain	Reference	Baratieri et al, 2013	Brodin et al, 1996	Hamersky et al, 1980	Khoshibin et al, 2019	Lazzaretti et al, 2014	Monardes et al, 2018	Perinetti et al, 2004	Perinetti et al, 2005	Sabuncuoglu and Ersahan 2014b	Sabuncuoglu and Ersahan 2016	Veberiene et al, 2009	Villa et al, 2005
1. Confounding	1.1	PY	PY	PY	PY	PY	PY	PY	PY	PY	PY	PN	PY
	1.2	PN	N	N	N	N	PN	N	N	N	N	-	N
	1.3	-	-	-	-	-	-	-	-	-	-	-	-
	1.4	PN	N	PN	N	N	PN	PN	PN	PN	PN	-	PN
	1.5	-	-	-	-	-	-	-	-	-	-	-	-
	1.6	NI	NI	NI	NI	NI	NI	NI	NI	NI	NI	-	NI
	1.7	NI	N	NI	NI	NI	NI	NI	NI	NI	NI	-	NI
	1.8	-	N	-	-	-	-	-	-	-	-	-	-
	Judgement	Serious	Serious	Serious	Serious	Serious	Serious	Serious	Serious	Serious	Serious	Low	Serious
2. Selection of participants into the study	2.1	N	PY	N	N	PY	PN	N	N	N	N	N	N
	2.2	-	PY	-	-	PY	-	-	-	-	-	-	-
	2.3	-	PY	-	-	PY	-	-	-	-	-	-	-
	2.4	Y	Y	Y	Y	Y	PY	PY	PY	PY	PY	PY	Y
	2.5	-	PN	-	-	PN	-	-	-	-	-	-	-
	Judgement	Low	Critical	Low	Low	Critical	Low	Low	Low	Low	Low	Low	Low
3. Classification of interventions	3.1	Y	Y	Y	Y	Y	PN	Y	Y	Y	Y	Y	Y
	3.2	PY	PY	PY	PY	PY	PY	Y	Y	Y	Y	Y	Y
	3.3	PN	PN	PN	PN	PN	PY	PN	PN	PN	PN	PN	PN
	Judgement	Low	Low	Low	Low	Low	Moderate	Low	Low	Low	Low	Low	Low
4. Deviations from intended interventions	4.1	N	N	N	N	N	N	N	N	N	N	N	N
	4.2	-	-	-	-	-	-	-	-	-	-	-	-
	4.3	Y	Y	Y	NI	PY	NI	PY	PY	Y	Y	Y	PY
	4.4	Y	Y	Y	Y	Y	Y	Y	Y	Y	Y	Y	Y
	4.5	Y	Y	PY	PY	Y	PY	PY	PY	Y	Y	Y	PY
	4.6	-	-	-	-	-	-	-	-	-	-	-	-
	Judgement	Low	Low	Low	Moderate	Low	Moderate	Low	Low	Low	Low	Low	Low
5. Missing data	5.1	PY	N	Y	PY	PN	PY	PY	PY	Y	Y	PY	PY
	5.2	N	N	N	N	N	PN	PN	PN	PN	PN	NI	NI
	5.3	NI	N	N	N	N	NI	NI	NI	N	N	NI	NI
	5.4	-	NI	-	-	NI	-	-	-	-	-	-	-
	5.5	-	NI	-	-	NI	-	-	-	-	-	-	-
	Judgement	Moderate	Serious	Low	Low	Serious	Moderate	Moderate	Moderate	Low	Low	Moderate	Moderate
6. Measurement of outcomes	6.1	N	PN	NI	NI	N	NI	PN	PN	PN	PN	PN	N
	6.2	N	NI	NI	NI	N	NI	NI	NI	NI	NI	NI	N
	6.3	Y	Y	Y	Y	Y	PY	PY	PY	PY	PY	PY	Y
	6.4	PN	PN	PN	PY	PN	NI	NI	NI	PN	PN	PN	PN
	Judgement	Low	Moderate	Moderate	Serious	Low	Serious	Moderate	Moderate	Moderate	Moderate	Moderate	Low
7. Selection of the reported result	7.1	NI	NI	NI	NI	NI	NI	NI	NI	NI	NI	NI	NI
	7.2	NI	NI	NI	NI	NI	NI	NI	NI	NI	NI	NI	NI
	7.3	NI	NI	NI	NI	NI	NI	NI	NI	NI	NI	NI	NI
	Judgement	Moderate	Moderate	Moderate	Moderate	Moderate	Moderate	Moderate	Moderate	Moderate	Moderate	Moderate	Moderate
Overall	Judgement	Serious	Critical	Serious	Serious	Critical	Serious	Serious	Serious	Serious	Serious	Moderate	Serious

Y, yes; N, no; PY, probably yes; PN, probably no; NI, no information

Orthodontic tooth movement has been characterised as an inflammatory process. In essence, following force application of various levels, bone remodeling occurs through bone resorption and apposition in the direction of tooth movement and against it, respectively.^29^ The tooth and supporting tissue apparatus may experience transient or more permanent alterations, while it has been documented that the optimum force levels for orthodontic tooth movement are expected to stimulate cellular activity without occluding the blood vessels and without cutting off the blood supply within the PDL.^10^ Depending on the type and magnitude of orthodontic movement, the periodontal apparatus experiences different types of stress. Specifically, forces that do not present a uniform compression surface within the PDL are more likely to lead to hyalinisation or increase the risk of root resorption or loss of pulp vitality.^33,44,52^

Pulp response to orthodontic forces during the initial stages of tooth movement and in the short term may begin with hemodynamic changes^38,49^ and circulation disturbance,^6,53^ with an additional increase in the density of blood vessel volume.^16,17^ An increase of neural activity with the local release of neuropeptides has been detected,^41,42^ which subsequently leads to changes in the pulp metabolism, resulting in apoptosis and necrosis of pulp cells. The resulting presence of macrophages, changes of the odontoblast layer, edema, and an increase of fibroblasts and progenitor cells may indicate inflammation and adaption of the pulpal tissue caused by orthodontic forces.^4^

Nevertheless, discussion is still ongoing about the long-term effect of orthodontic forces on pulp tissues. Lazzaretti et al^31^ reported that heavy and continuous forces may lead to an increased potential for the formation of pulpal calcifications. Although pulpal calcifications are commonly seen in patients and are age-dependent, extensive pulpal calcifications or even total calcifications of the root pulp have been observed in patients with a history of orthodontic treatment. However, a recent prospective controlled clinical study trial conducted by Baratieri et al^5^ did not support the theory of newly formed pulp chamber calcification induced by orthodontic force application.

Although the aforementioned changes within the pulp occur, there is a deficit of high-quality studies to support the connection between orthodontic force application and pulp tissue reactions. Previously published systematic reviews in the broader field^15,27,56-58^ have limitations for different reasons: they are outdated, focus solely on certain outcomes and particular use of specific instrumentation to detect pulpal response, are solely related to specific types of tooth movement, or comprise qualitative syntheses of studies whose design cannot support any aetiologic associations of the effect-outcome interrelation.

Therefore, the present systematic review (SR) is considered timely and justified. Its aim was to determine any changes of the pulp tissue or alteration in response to the application of orthodontic forces. It consists exclusively of prospectively designed clinical trials (either randomised or not), and includes outcomes at all levels, e.g. histologic alterations, blood flow, pulp volume changes, pulp calcification, and pulp necrosis.

## Methods

### Protocol and Reporting

The protocol of this systematic review was developed a priori and after implementation of the search strategy, and is now registered in the Open Science Framework [https://osf.io/avfhb/]. The review was reported according to the latest PRISMA 2020 reporting guidelines.^40^

### Search Strategy

An electronic search of the published and unpublished literature was conducted on 14 February 2021 and updated on 1 August 2022, separately and by two examiners (IS, DK). The main formal databases were MEDLINE via Pubmed, Scopus, Cochrane Central (CENTRAL), and the Cochrane Database for Systematic Reviews (CDSR) and LILACS (Virtual Health Library: https://lilacs.bvsalud.org/en/). Unpublished reports were sought through ClinicalTrials.gov (www.clinicaltrials.gov) and the National Research Register ISRCTN (www.controlled-trials.com). Hand searching was conducted in the retrieved for full-text evaluation articles for any additional potential for inclusion in this publication. No filters were used. The entire search strategy is presented in Appendix 1.

### Eligibility Criteria

- Study design: randomised controlled trials (RCTs) and prospective clinical trials were included in the review.
- Participants: all patients undergoing orthodontic treatment with any type of appliance/method. No age or gender restriction was applied.
- Intervention: any type of orthodontic appliance, as defined by the authors of the primary studies.
- Comparators: non-treated control, or any other type of orthodontic treatment as a comparator.
- Outcome: outcomes at all levels of pulp reactions were included, namely, but not limited to: histologic alterations, blood flow, pulp volume changes, pulp calcification, and pulp necrosis. All movement types were considered (also surgically accelerated tooth movement studies, i.e. piezocision/corticotomy, etc).
- Exclusion criteria: retrospective observational studies, and case reports/series. Single-arm trials without a comparator group – either control or other treatment/intervention – were excluded.

### Study Selection

Titles and abstracts of initially retrieved articles were screened independently by two reviewers (IS, DK). Full texts of potentially eligible articles were examined at a second stage by both reviewers; the final inclusion of articles was based on consensus after consultation with a third author (GNT) if/when discrepancies were identified at any point during the process.

### Data Collection Process

Data were extracted and recorded in pre-piloted forms. These forms included specific characteristics of the study and information on the study design, title, authors, date, population, interventions, comparators, and outcomes. Data were extracted by two reviewers (IS, DK) and re-examined by a third (GNT). Identified inconsistencies were settled after discussion until a consensus was reached.

### Risk of Bias in Individual Studies

Risk of bias assessment of individual studies was conducted according to their study design. For RCTs, the Cochrane RoB tool 2.0 was used.^51^ For non-randomised prospective controlled trials, the ROBINS-I tool was used (Risk of Bias in Non-randomised Studies of Interventions).^50^ Randomised trials were considered to be all studies reporting a random assignment of the interventions in the methodology section. Then, their quality/risk of bias was determined using the Cochrane RoB 2.0 tool. For the assessment and presentation of the risk of bias across studies, the web application tool Robvis was utilised.^34^

### Summary Measures and Data Synthesis

Quantitative synthesis of study findings was performed where possible, after exploring heterogeneity levels both clinically and statistically across individual reports. Statistical heterogeneity was first assessed visually through inspection of the confidence bounds within the forest plots; it was also statistically tested using I^2^, where p < 0.10 indicated non-homogeneity. Random effects meta-analyses were conducted in view of the potential, anticipated heterogeneity. Pooled estimates were presented if two or more studies were deemed eligible for a single comparison. Estimates were presented as mean differences (MDs) (effect sizes) with corresponding confidence bounds (95% CI) given the nature of the anticipated outcomes. Authors of the studies were contacted for additional data if not all available information was provided in the published document. As some of the studies included had a split-mouth design, the mean differences for those were calculated between quadrants and the standard deviation of the difference was approximated by the formula:


$$SD_{diff}=\sqrt{sd_{1}^{2}+sd_{2}^{2}-2rsd_{1}sd_{2}}$$

where sd_1_ and sd_2_ indicate the standard deviations in quadrants and r is the correlation coefficient between quadrants. The correlation coefficient was set at r = 0.5 for split-mouth studies and r = 0 for parallel designs.

### Risk of Bias Across Studies

Publication bias was planned to be explored through standard funnel plots and Egger’s regression test, if applicable.^18^

### Additional Analyses

Sensitivity analyses were considered, if applicable, to explore and isolate the effect of studies with a high risk of bias on the overall effect, if studies of both high or lower risk of bias were ultimately included in the quantitative synthesis. In addition, sensitivity analyses were considered if variability in population characteristics was identified across studies (i.e. age).

### Assessment of the Certainty of the Evidence

The Grading of Recommendations Assessment, Development and Evaluation (GRADE) was performed to assess the overall quality of the evidence, as formulated by the research question, interventions, comparators and outcomes for evaluation.^21^ According to GRADE, the overall body of evidence is rated as high, moderate, low and very low. Assessment of the body of evidence primarily involves an initial assessment of the study design (randomised or not). Assessment is made for the following the domains: risk of bias, inconsistency, indirectness, imprecision and publication bias. For RCTs, the first 4 domains of the quality of evidence may be downgraded on the basis of either ‘serious’ or ‘very serious’ risks (1 or 2 levels respectively); publication bias may either be suspected or undetected (1-level downgrade). For non-randomised/observational designs, which theoretically start from a ‘low’ level of evidence, the potential for upgrade is as follows: a large or very large effect, plausible residual confounding that may alter the effect, or a dose-response gradient. The level of evidence may be upgraded by 1 or 2 levels (large effect), or 1 level (plausible confounding, dose-response gradient).

## Results

### Search Details

**Fig 1:**
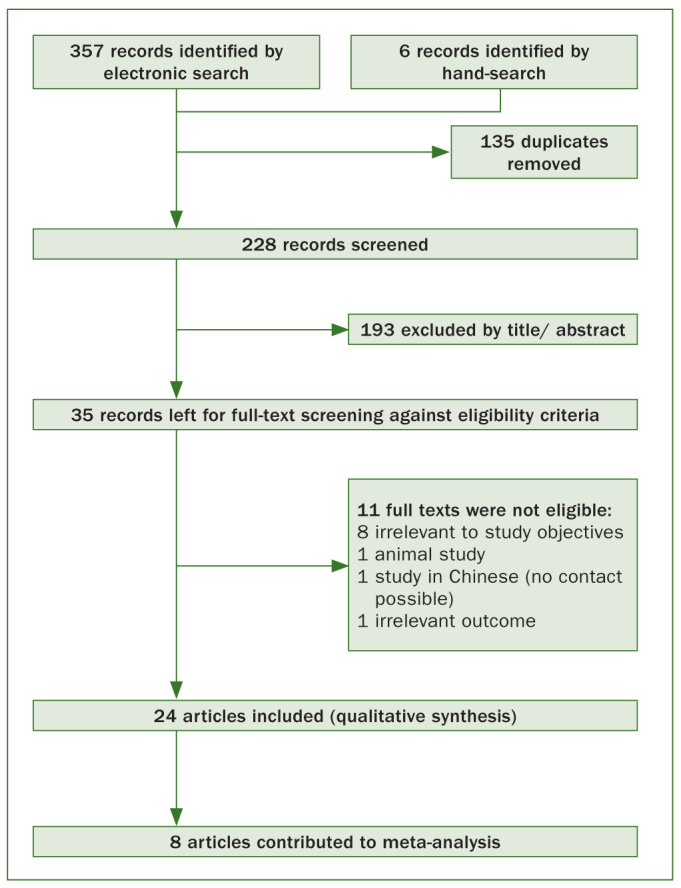
Flow diagram of study selection and inclusion.

Eligibility and selection of studies for qualitative and quantitative synthesis is presented in Fig 1. From an initial number of 363 results, 35 articles passed through full-text assessment process, and 24 were ultimately deemed eligible for inclusion in the qualitative synthesis. Eight of these qualified for the meta-analysis.

### Study Design and Characteristics

Twelve studies were randomised controlled trials,^1-3,11,12,19,23-25,37,43,46^ and 12 more were non-randomised prospective clinical trials.^5,9,22,28,31,36,41,42,47,48,54,55^ All characteristics of the included studies – design, interventions, comparators, and outcomes assessed – are shown in Table 1. The split-mouth design was more prevalent, being identified in 14 of 24 studies.

**Table 1:** Characteristics of included studies (RCT: randomised controlled trial; nR-PCT: non-randomised prospective clinical trial)

Study	Population	Intervention	Comparison	Outcome	Notes
Abu Alhaija et al, 2019, RCT	45 patients (28 female, 17 male) (all teeth)	Experimental group n = 25 Age: mean 18.77 ± 1.13 years Preadjusted edgewise fixed appliance with 0.014 NiTi Type of force: standard alignment	Comparator group n = 20 Age: mean 19.04 ± 1.61 years Clear aligners (until after delivery of 2nd aligner)	Pulpal blood flow (PBF) changes measured by a laser doppler flowmeter (LDF)	Observation timepoints: 20 min 48 h 72 h 4 weeks
Abu Alhaija and Taha, 2021, RCT (split-mouth)	22 patients (16 female, 6 male) (all teeth) Age: mean 19.0 ± 2.53 years	Experimental group n = 22 Self-ligating brackets 0.016 NiTi 0.016 x 0.022 NiTi Type of force: standard alignment	Comparator group n = 22 Conventional brackets 0.016 NiTi 0.016 x 0.022 NiTi	Pulpal blood flow (PBF) changes measured by a laser doppler flowmeter (LDF)	Observation timepoints: 20 min 48 h 72 h 4 weeks
Al-Ainawi et al, 2016, RCT (split-mouth)	7 patients (16–25 years), (14 canines) No information on the gender Inclusion criteria: bilateral extraction, no abnormal position of the canines, no endodontic treatment	Experimental group n = 7 Osteotomy (line mesial and distal to the canine) and extraction of the PM at the same appointment 2 different distractors (modified has a hook), this arm was adapted to enter the osteotomy line at a mesial point to the canine Distraction 1 mm per day (start 5 days after the operation) After the canine has reached the desired position, the distraction device was kept passive for 1 month Pulp vitality of the canine tested with an electrical pulp tester immediately pre-distraction, immediately post-distraction and 3 months post-distraction	Control group n = 7 Normal distractor	Pulp vitality (all 14 canines were vital before the study, none reacted positively to the vitality test after completion of the distraction, in each group, 4/7 were vital following the distraction completion) No discoloration or pulpal pain Short follow-up (3 months)	Surgical procedure done by 1 dentist, orthodontic therapy was performed by postgraduate students
Baratieri et al, 2013, nR-PCT	30 patients (60 maxillary molars and 60 central incisors) 18 boys (mean age 9.7 years) 12 girls (mean age 9.4 years)	Treatment group: 15 patients; 8 boys and 7 girls (mean age 9.6y), 30 molars and 30 incisors Soft-tissue borne Haas-type appliance; SS wire diameter of 0.047 inch and expansion screw of 11 mm. Banded on the first permanent and first deciduous molars. 0.5 mm activation per day. Retention for 6 months CBCT before and 1 year after the rapid maxillary expansion (i-CAT, 120 kV, 5mA, FOV 16x22cm) Scans were randomly selected and the examiner was blinded	Control group: 15 patients; 10 boys and 5 girls (mean age 9.4 years) 30 molars and 30 incisors Comparison of pulp size and appearance of pulp stones between the 2 groups	Pulp stones: no new pulp chamber calcification was found in either group Chamber dimension: 1 year after expansion, incisor pulp dimensions were minimally wider, whereas in the untreated group, they were reduced	
Brodin et al, 1996, nR-PCT (split-mouth)	6 healthy dentistry students (10 lateral incisors) No information on gender or age	Treatment group n = 5 Brackets were bonded on the labial surface of 3+3 and the laser doppler flowmetry probe was inserted into a steel tube bonded to the labial surface of the lateral incisors at a distance of 2 mm from the gingival margin 0.016 x 0.022 stainless steel sectional attached on 3+3 Lingual buttons 2+2 2-Newton intrusive or extrusive force was applied for 5 min Stabilization of blood flow for 30 min between each test	Control group: n = 5 the lateral incisor on the contralateral side served as a control Comparison of blood flow after extrusion or intrusion	Pulpal blood flow changes measure by laser doppler flowmetry: Extrusion: no changes in the pulpal blood flow Intrusion: immediate reduction of the pulpal blood flow to 80% of the pretreatment values Unloading contributed to the increase in blood flow which returned to the control level after 3 min	Outcome change measured as a percentage of pretreatment
Caviedes-Bucheli et al, 2021, RCT (split-mouth)	20 patients (12 female, 8 male) (40 premolars) Age: range 18–30 years	Experimental groups 1. Occlusal trauma n = 5 (2 teeth/patient) 2. Orthodontic force (56 g extrusive force) for 24 h n = 5 (2 teeth/patient) 3. Occlusal trauma+ orthodontic force n = 5 (2 teeth/patient)	Control group n = 5 (2 teeth / patient) No Intervention	Expression: substance P, calcitonine gene-related peptide (CGRP), vascular endothelial growth factor (VEGF) Radioimmunoassay	
Chavarria et al, 2014, RCT (split-mouth)	8 patients (16 maxillary premolars) 12–16 years Both genders, no further information	Experimental group n = 8 premolars Intrusion for 24 h (force 150–200g) 0.018-inch brackets were bonded on the first molar and first premolar. Sectional (0.018 x 0.025 stainless steel with angulation of 40° After the extraction: acquisition of the pulpal tissue and storage in 4% paraformaldehyde	Control group: n = 8 premolars The contralateral premolar of each patient served as control, same procedure as the experimental group, but without orthodontic force had been applied Comparison of the expression of SP, CGRP, B-end and Met-Enk after Intrusion vs no force	SP, CGRP, B-end and Met-Enk RIA (radioimmunoassay) SP and CGRP levels in experimental group support the positive correlation between the symptomatic clinical scenario and increased expression levels of neuropeptides, clarifying the role of neurogenic inflammation in the early injury response low increasing tendency observed in the opioid levels illustrates a poorly established pain-modulating system during the first 24 h of intrusion	SP = Substance P: mediator of neurogenic inflammation causes vasodilatation CGRP = calcitonin gene-related peptide: vasodilatory peptide B-End and Met-Enk: Opiod peptides
Ersahan and Sabuncuoglu 2015, RCT (split-mouth for control)	20 patients (20 maxillary molars) Age: mean 27.6 years (range 20–40 years)	Experimental groups 1. n = 10, force 125 g 2. n = 10, force 250 g 0.016 x 0.022 stainless steel wire was placed in the maxillary arch in preparation for mini-implant insertion Intrusive forces (through elastic power chains) to over-erupted molars Measurements were recorded at 1, 3, and 7 days, 3 and 4 weeks, and 3 and 6 months after intrusion. Intrusion was accomplished in 6 months	Control group n = 10 Contralateral No orthodontic force	Pulpal blood flow (PBF) changes measured by a laser doppler flowmeter (LDF)	
Hamersky et al, 1980, nR-PCT (split-mouth)	17 patients (12 female, 5 male) (68 premolars) Age: 15 years (range 11.8–25.8 years)	Experimental group n = 34 Bracket placement lingual to the maxillary first premolar and buccal to the mandibular first premolar Type of force: extrusive Elastics for 72 h (1/8 inch 6-ounce) 24 h a day, except during meals and oral hygiene	Control group n = 34 The contralateral premolar of each patient served as control, same procedure as the experimental group, but no orthodontic force was applied	Pulpal respiration 17 subjects showed pulp tissue respiration which was depressed an average of 27%. Positive correlation between patients age and amount of tissue respiratory depression	After extraction: dissection with a high-speed handpiece (groove 1 mm) and a surgical chisel. Measurement of pulp weights Radioactively labeled carbon dioxide production system to evaluate the effect of the orthodontic force on pulpal respiration
Han et al, 2013, RCT	27 patients (12 female, 15 male) (54 maxillary premolars) Age: mean 17.9 years (range 14–24 years)	Experimental groups 1. n = 12 patients, moderate force 50 g 2. n = 12 patients, severe force 300 g Intrusive orthodontic forces were applied by a clear, closed elastomeric chain The forces were applied for 1, 4, 8, or 12 weeks	Control group n = 3 patients	Pulp vitality: electric pulp and thermal tests Histologic outcomes: Odontoblastic degeneration Vacuole formation Vascular dilation Pulp stones	
Han et al, 2020, RCT	15 patients (30 maxillary premolars) No information on gender/ age	Experimental groups 1. n = 6 force applied for 1 week 2. n = 6 force applied for 4 weeks 3. n = 6 force applied for 8 weeks 4. n = 6 force applied for 12 weeks An intrusive force of 300 g was applied by an elastic rubber band	Control group n = 6 No orthodontic force	Expression of c-FOS and MMP-9	Protein expression of c-FOS and MMP-9 may be related to molecular changes in the cells of the dental pulp
Hatrom et al, 2021, RCT	23 patients Age range 15–26 years Inclusion criteria: bilateral first premolar extraction in the maxilla	12 patients in the piezosicion (1 dropout), 7 males, 6 females, age 19.27 years ( ± 3.38) Modified bidimensional bracket system: 0.018 inches in the anterior, 0.022 inches in the posterior En masse retraction after 1 week on a 0.018 x 0.025 inch SS wire through NiTi active coil springs (force 250 grams)	11 patients the control group (2 dropouts), 6 males, 7 females, age 20.83 years ( ± 3.64 years)	Change in pulp volume (maxillary anterior teeth canine to canine) Root resorption	CBCTs taken during the initial visit and at the end of the en masse retraction Short duration of the study did not allow sufficient time for secondary dentin to form
Khoshibin et al, 2019, nR-PCT	129 patients, 516 teeth (maxillary central and lateral incisors) 0.012 NiTi group: 21.2 ± 6.7 years 0.014 NiTi group: 23.6 ± 9.5 years Control group: 23.8 ± 9.1 years No information on the gender	2 experimental groups 0.012 NiTi archwire, n = 43 0.014 NiTi archwire, n = 43 MBT straight wire technique, 0.022 slot Force type: alignment EPT (electric pulp test) was performed 3 times: 1. pre-bonding (EPT0) 2. immediately upon initiation (5 min) (EPT1) 3. 1 month post-therapy (EPT2)	Control group n = 43 Similar EPT testing	Response on EPT All teeth gave positive responses at EPT0 and EPT1, 12% failed at EPT2	False positive and false negative responses are commonly encountered with EPT
Lazzaretti et al, 2014, nR-PCT (split-mouth)	17 patients (34 maxillary first premolars) Age: 12–19 years 9 female, 8 male	Experimental group n = 17 Sensitivity test and radiograph before and after the orthodontic appliance Intrusion force (60 g) for 21 days 0.019x0.025 SS Sectional from 1st molar to 1st premolar	Control group n = 17	Histologic outcomes based on optical microscope classification: inflammatory response, soft and hard tissue response, vascular alterations	Patients with first premolars whose side of the mouth presented less crowding were selected for the experimental group [sic] After extraction, teeth were fixed in 10% formaldehyde, processed, and histotechnically prepared
Monardes et al, 2018, nR-PCT	37 patients (136 teeth: 63 rotation, 73 sagittal movement) No information on age/gender	Experimental group: 136 teeth 0.014 inch heat activated NiTi 4 types of force levels applied: (i) extreme (>100 g), high (76–100 g), (iii) optimal (26–75 g), and (iv) mild (0–25 g)	Control group: number not reported	Pulp response to hot/ cold stimuli (sensitivity tests): No response Normal response Increased response	Pulp sensitivity examined on the 7th day after force application/similarly to control
Mostafa et al, 1991, RCT (split-mouth)	18 patients (36 maxillary 1st premolars) Mean age 18 years (range 16–21 years) No information on the gender	Experimental group n = 18 Fixed orthodontic appliance with an activated spring attached Extrusion due to: 1. extraction after 1 week of activation 2. extraction after 2 weeks of activation 3. extraction after 4 weeks of activation Mean force: 1st day 57 g/ 1st week 54 g/ 2nd week 52 g/ 4th week 48 g	Control group n = 18	Histologial outcomes: vacuolization of the pulp tissues, congested blood vessels, odontoblastic degeneration, pulp fibrosis	No numerical data presented
Perinetti et al, 2004, nR-PCT (split-mouth)	17 patients (34 maxillary 1st premolars), 11 female, 6 male Age: mean 16.8 ± 1.6 years	Experimental group n = 17 Orthodontic brackets were placed on the buccal surfaces of all teeth in the maxillary arch Force: 30–90 grams Type of movement: alignment 7 days of treatment and extraction	Control group n = 17 No force application	Aspartate aminotransferase activity (AST) measured with a spectrometer	AST activity is increased in reversible pulpitis and decreased in irreversible pulpitits Significantly higher AST activity in the experimental group (6.7 ± 1.9 U/mg of pulp tissue In control teeth, the enzymatic activity was 3.6 ± 1.4 U/mg of pulp tissue
Perinetti et al, 2005, nR-PCT (split-mouth)	16 patients (32 maxillary 1st premolars), 10 female, 6 male Age mean: 17.0 ± 1.6 years	Experimental group n = 16 Orthodontic brackets were placed on the buccal surfaces of all teeth in the maxillary arch Force: 30–90 grams Type of movement: alignment 7 days of treatment and extraction	Control group n = 17 No force application	Alkaline phosphatase activity (ALP)	ALP is an enzyme involved in tissue mineralisation and periodontal tissues Significantly lower ALP activity in the experimental group 89 ± 26 U/(L x mg) In the control group, the enzymatic activity was 142 ± 33 U/(L x mg) The decrease in ALP activity in the experimental group might be explained by damage of the pulp cells responsive to the synthesis of this enzyme.
Ramazanzadeh et al, 2008, RCT (split-mouth/ not clear design)	26 patients (52 maxillary 1st premolars), 16 female, 10 male Age: mean 18 ± 3.2 years	Experimental groups n = 40 10 teeth were intruded for 3 days 10 teeth were intruded for 3 weeks 10 teeth were extruded for 3 days 10 teeth were extruded for 3 weeks 0.018 inch standard edgewise brackets bonded to the premolar Extrusion: 1/4 inch light elastics (from the maxiillary to the mandibular premolar, force ~ 75 g, worn for 24 h) Intrusion: NiTi coil spring (0.010 x 0.030 inch, force 25 ± 5 g) Extraction at 3 days and 3 weeks Light microscope examination	Control group n = 12	Histological outcomes: acute or chronic inflammation of the pulp, fibrotic tissue formation, necrosis, disruption of odontoblastic layer, odontoblast aspiration into the dentin tubules, formation of reparative dentin, pulp stones, dilation in vessel diameters, vacuole formation in the odontoblastic layer, resorption of dentin/cementum	Lack of difference between intrusion and extrusion and no vascular dilatation resorption might be due to light forces Odontoblastic aspiration can be the first visible pulp reactions to external stimulus. It could be regarded as a result of the extraction.
Sabuncuoglu and Ersahan 2014a, RCT (split-mouth for control)	20 patients (40 teeth) Anterior teeth intrusion Age: mean 20.3 years (range: 18–25 years) Deep bite (4 mm) No information on gender	Experimental groups n = 20 (40 teeth): Group 1 (n = 10/ 20 teeth) 40 g intrusive force maxillary left central and lateral incisors Group 2 (n = 10/ 20 teeth) 120 g intrusive force maxillary left central and lateral incisors 0.018 x 0.025 brackets were bonded and the teeth were leveled (segmental arch) until a 0.016x0.022 stainless steel wire could be placed Mini-implant between the roots of the maxillary left central and lateral incisors Intrusion (NiTi springs) for 3 weeks at the experimental teeth	Control group n = 40 contra-lateral incisors No orthodontic treatment	Pulpal blood flow (PBF) changes measured by a laser doppler flowmeter (LDF)	Observation at 3 days and 3 weeks LDF assessment of PBF is highly susceptible to environmental and technical factors
Sabuncuoglu and Ersahan 2014b, nR-PCT	16 patients (32 maxillary molars) Molar intrusion Age: mean 21.7 years (range 18–25) Anterior open bite (more than 2mm) No information on the gender	Experimental group n = 10/ 20 teeth Intrusion with elastic power-chains 100 g 4 mini-implants (2 per tooth) 0.016 x 0.022 stainless steel wire	Control group n = 6/, 12 teeth No orthodontic treatment	Pulpal blood flow (PBF) changes measured by a laser doppler flowmeter (LDF)	Observation 3 days 3 weeks 3 months 6 months At 3 and 6 months, the PBF rebounded to original
Sabuncuoglu and Ersahan 2016, nR-PCT	24 patients (14 female, 10 male), 48 maxillary premolars (2 from each patient), non-split-mouth design Age: mean 21.91 ± 2.89	Experimental group n = 12 (24 teeth) 0.016x0.022-inch stainless-steel Mini-implant use Orthodontic force 100 g through NiTi closed-coil springs Canine retraction accomplished in 4 months	Control group n = 12 (24 teeth)	Pulpal blood flow (PBF) changes measured by a laser doppler flowmeter (LDF)	Observation timepoints: 24 h 3 days 1 week 4 weeks 16 weeks (end of retraction period)
Veberiene et al, 2009, nR-PCT (split-mouth)	21 patients (42 premolars) Intrusion Age: mean 15.5 ± 0.5 years No information on the gender	Experimental group n = 21 Spring (0.016x0.022 stainless steel) Mean intrusion force 61 g Removal of the spring after 7 days and electrical stimuli through ‘Pulptester’ (Lumen; Kaunas, Lithuania)	Control group n = 21 Electrical stimuli with ‘Pulptester’ (Lumen; Kaunas, Lithuania), pulp testing prior to extraction	Aspartate aminotransferase activity (AST, measured with a spectrometer) Sensitivity with electrical pulp testing (EPT)	
Villa et al, 2005, nR-PCT	25 patients (50 maxillary 1st premolars), 13 female, 12 male Age range: 12–45 years	Experimental group n = 34 Right PM: Placebo, 2 pills per day every 24 h for 7 days Left PM: NSAID (nabumetone), 500 mg every 24 h for 7 days Treatment time 8 weeks 0.017x0.025 stainless steel intrusive force of 113 g	16 teeth served as control, no orthodontic force was applied Comparison of inflammatory changes of the pup-dentin complex, appearance of root resorption, tooth pain and tooth movement in patients given a dose of an NSAID	Pulp-dentin complex changes: collagen fiber density and quality pulp angiogenesis odontoblast layer vacuolisation External root resorption, degree and amount	Histological sections divided into 3 zones: coronal, middle and apical thirds

The total number of participants ranged from 6 to 129 patients, with an age varying from an average of 9.4 years to a maximum of 45 years old. The number of included teeth per study was 10 to 516, and the most frequently studied teeth were premolars (13/24; 54.2%). Of the 24 studies, 21 compared the intervention with a control group with no application of any orthodontic force, while in three studies, the experimental group was compared with a group of patients receiving other interventions (i.e. conventional brackets vs self-ligating, conventional brackets vs orthodontic aligners, use of different types of distractors for osteotomy-mediated tooth movement acceleration). Regarding the type of tooth movement assessed, intrusion of various force levels was attempted in 11 of 24 studies (45.8%), extrusion in 3 (12.5%), alignment in 5 (20.8%) and retraction in 2 (8.3%). The most prevalent type of outcome assessed were changes in pulpal blood flow, determined in 7 studies (29.2%), which in all cases was measured by laser doppler flowmetry. Other outcomes pertained to activity and expression of peptides and enzymes, such as aspartate aminotransferase (AST), alkaline phosphatase (ALP), substance P, calcitonin gene-related peptide (CGRP), as well as histologic outcomes and others. Timing for outcome evaluation differed across the studies, ranging from 20 min following force application to 6 months overall (Table 1).

### Risk of Bias Within Studies

**Fig 2:**
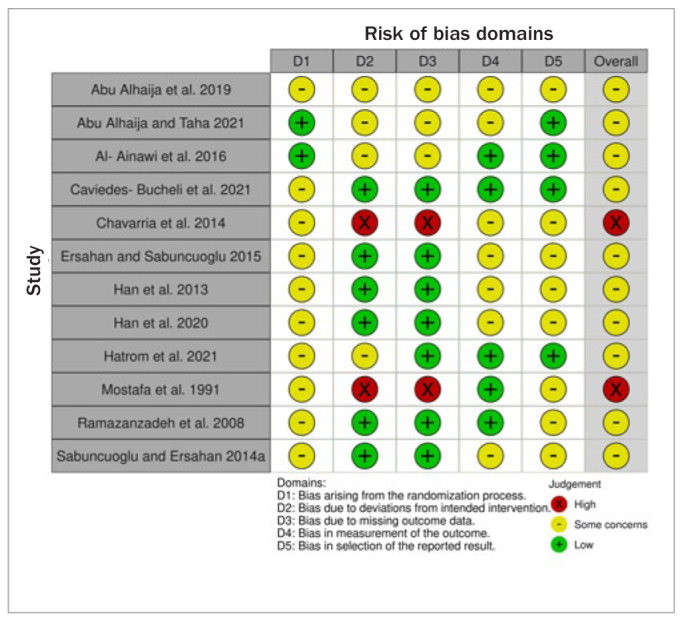
Risk of bias summary graph across studies [RoB 2.0 tool for Randomized Controlled Trials, RCTs].

The risk of bias for the RCTs included in the present SR ranged from ‘raising some concerns’ to ‘high overall’. The latter rating was mainly due to missing data and potential for deviations from the intended intervention in two of the included studies. Some concerns were raised for the rest of the studies as well, mainly due to inadequate reporting of the randomisation process, no information on the masking of outcome assessors involved, or non-existence of a pre-registered protocol, thus not precluding a potential selection of the presented results (Fig 2; Supplementary Table 1).

**Fig 3:**
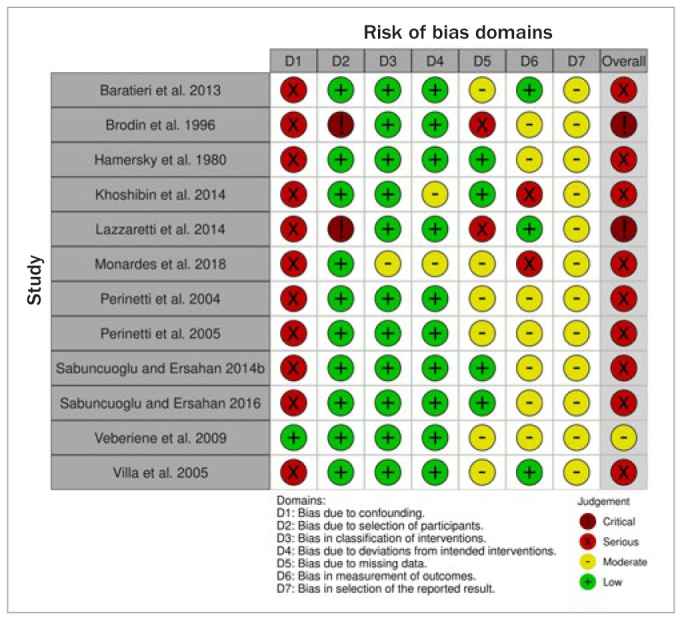
Risk of bias summary graph across studies [ROBINS-I tool for non-Randomized Prospective Clinical Trials Trials, nR-PCTs].

For the nR-PCTs, the risk of bias ranged from ‘moderate’ to ‘critical overall’, with most studies presenting serious risk of bias. In almost all cases, there were concerns about the effect of potential confounding factors that had not been adequately addressed and considered. Again, in all but three studies, there was a potential for moderate to serious risk of bias due to inadequate reporting of measurement of the outcome, in terms of masking the evaluator. Last, the risk for selection of the reported result could not be precluded, since none of the included nR-PCTs reported the existence of a registered protocol (Fig 3; Supplementary Table 2).

### Effects of Interventions, Meta-Analysis and Additional Analyses

A total of 8 studies contributed to the meta-analysis, being combined in different syntheses according to the respective outcomes and population characteristics.

**Fig 4:**
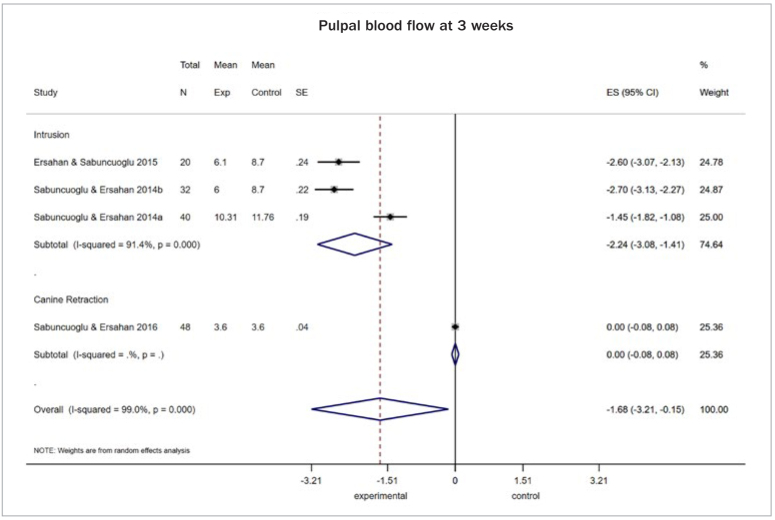
Random effects meta-analysis for summary mean difference [with 95% CI] in pulpal blood flow (PBF) in perfusion units, in teeth subjected to orthodontic tooth movement for 3 weeks under a force of 100-120 g, compared to no force application.

**Fig 5:**
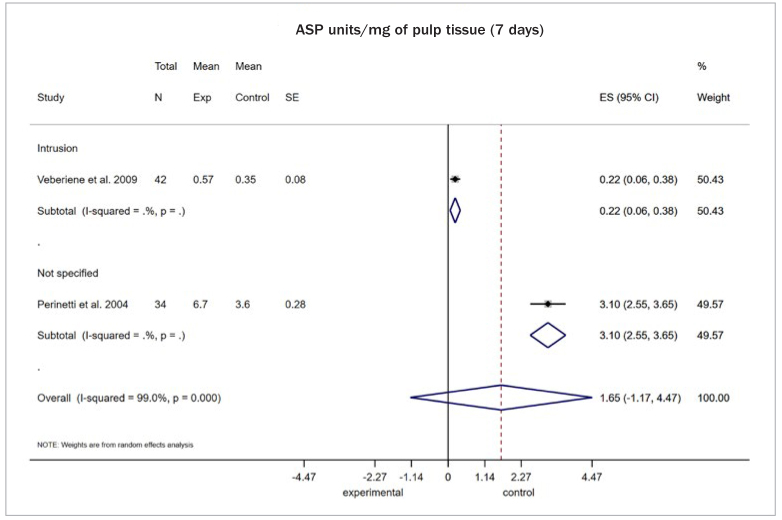
Random effects meta-analysis for summary mean difference (with 95% CI) in aspartate aminotransferase (AST) activity at 7 days (in units/mg of pulp tissue), in teeth subjected to orthodontic tooth movement under a force of 30–90 g, compared to no force application.

For pulpal blood flow (PBF) assessed at 3 weeks in perfusion units, evidence was found of a decrease in teeth subjected to tooth movement (both intrusion and canine retraction) upon application of forces ranging from 100 to 125 grams, compared to non-orthodontically treated teeth (4 studies, MD: -1.68; 95% CI: -3.21, -0.15; p = 0.03; I2: 99.0%; Fig 4). However, this was not the case for molars assessed 6 months after intrusive forces of similar magnitude (2 studies, MD: -0.06; 95%CI: -0.36, 0.23; p = 0.68; I2: 0.0%). Furthermore, when use of conventional brackets was compared to other modalities (i.e. self-ligating or aligners) in terms of alignment movements, there was no evidence of any difference after 4 weeks of treatment, again when PBF was considered (2 studies, MD: 0.11; 95%CI: -0.31, 0.53; p = 0.60; I2: 50.1%). When AST activity was considered (in units/mg of pulp tissue), there was scarce evidence of a rising tendency at 7 days of assessment after application of light forces up to 90 grams (2 studies, MD: 1.65; 95%CI: -1.18, 4.47; p = 0.25; I^2^: 99%; Fig 5). Similarly, estimates from a single study confirmed a decreasing tendency in the recorded activity of ALP at 7 days (1 study, MD: -53.0; 95%CI: -67.70, -38.30; p < 0.001) (Table 2).

**Table 2:** Results of meta-analysis and single study estimates on outcomes related to the effects of orthodontic tooth movement to pulp

Synthesis	Number of studies	Mean difference	95% CI	p-value	I^2^ (%)	Tau-squared
Pulpal blood flow (in perfusion units) at 3 weeks of movement Movement vs no movement (force: 100–125 g)	4^a^	-1.68	-3.21, -0.15	0.03	99	2.39
Pulpal blood flow (in perfusion units) at 6 months of molar intrusion Movement vs no movement (force: 100–125 g)	2^b^	-0.06	-0.36, 0.23	0.68	0	0
Pulpal blood flow (in perfusion units) at 4 weeks of alignment Conventional brackets vs other (self-ligating or aligners)	2^c^	0.11	-0.31, 0.53	0.60	50.1	0.05
Aspartate aminotransferase (AST) activity at 7 days (in units/mg of pulp tissue) Movement vs no movement (force: 30–90 g)	2^d^	1.65	-1.18, 4.47	0.25	99	4.1
Alkaline phosphatase (ALP) activity at 7 days (in units/ltr*mg of pulp tissue) Movement vs no movement (force: 30–90 g)	1^e^	-53.0	-67.70, -38.30	<0.001	–	–
Pulp volume change at the end of en-masse retraction phase Piezocision vs no piezocision	1^f^	-0.05	-0.58, 0.48	0.85	–	–
Pulp respiration at 3 days Extrusion Movement vs no movement (force: 170 g)	1^g^	-6.60	-7.20, -6.10	<0.001	–	–
Expression of substance P at 24 h Intrusion Movement vs no movement (force: 150–200 g)	1^h^	48.40	32.33, 64.47	<0.001	–	–
Expression of calcitonin gene-related peptide (CGRP) at 24 h Intrusion Movement vs no movement (Force: 150–200 g)	1^h^	2.30	0.38, 4.22	<0.001	–	–
Expression of substance P on 24 h Extrusion Movement vs no movement (force: 56 g)	1^i^	0.65	0.64, 0.66	<0.001	–	–
Expression of calcitonin gene-related peptide (CGRP) at 24 h Extrusion Movement vs no movement (Force: 56 g)	1^i^	0.038	0.036, 0.040	<0.001	–	–

^a^ Ersahan and Sabuncuoglu 2015; Sabuncuoglu and Ersahan 2014a; Sabuncuoglu and Ersahan 2014b; Sabuncuoglu and Ersahan 2016

^b^ Ersahan and Sabuncuoglu 2015; Sabuncuoglu and Ersahan 2014b

^c^ Abu Alhaija et al 2019; Abu Alhaija and Taha 2021

^d^ Veberiene et al 2009; Perinetti et al 2004

^e^ Perinetti et al 2005

^f^ Hatrom et al 2021, ^g^ Hamersky et al 1980, ^h^ Chavarria et al 2014

^i^ Caviedes-Bucheli et al 2021

Further evidence from single-study estimates confirmed a relative decrease in the pulp respiration capacity after application of heavy extrusive forces for 3 days, compared to no treatment (1 study, MD: -6.60; 95%CI: -7.20, -6.10; p < 0.001). Upon application of both intrusive (150 to 200 grams) and extrusive (56 grams) forces for up to 24 h, there was strong evidence of increased expression of substance P and CGRP (p < 0.001 at all levels, based on single-study estimates) (Table 2).

Assessment of publication bias or the effect of high risk of bias in individual studies was not applicable, due to the scarcity of the existing reports (i.e. <10).

### Certainty of Evidence

The certainty of evidence for the outcomes assessed after data synthesis ranged from very low to low overall, based on a limited number of pooled studies. For PBF in the comparison of tooth movement with the absence of other force application, based on the combination of both RCTs and nR-PCTs, the certainty of evidence was rated as very low overall. Reasons for downgrading included risk of bias as well as limitations of the included studies and inconsistency. When PBF was rated comparing different types of orthodontic mechanics/interventions, a score of low certainty of evidence was achieved, and this included solely results from RCTs. Again, reasons for downgrading were study limitations and inconsistency. For AST, based on the synthesis of nR-PCTs, very low certainty of evidence was recorded due to study limitations, inconsistency, and imprecision in the estimated effect. No reason for an upgrade was detected (Table 3).

**Table 3:** Summary of findings table according to GRADE for all meta-analysis outcomes, showing number of studies, number of participants, effect estimates and quality of the evidence for pulpal blood flow (PBF) and aspartate aminotransferase (AST) activity

Outcomes related to pulp changes after orthodontic tooth movement						
Patient or population: Patients undergoing tooth movement Intervention: Tooth movement Comparison: No tooth movement/other intervention						
Outcomes*	Illustrative comparative risks* (95% CI)		Relative effect (95% CI)	No. of teeth/ participants (studies)	Quality of evidence (GRADE)	Comments
	Assumed risk	Corresponding risk				
	Control	Orthodontic force application				
Pulpal blood flow (in perfusion units) at 3 weeks of movement Movement vs no movement (force: 100-125 g)		The mean PBF in the intervention groups was 1.68 lower (3.21 lower to 0.15 lower)		140 (4 studies)	⊕⊖⊖⊖ very low^1,2^	Both RCTs and nR PCT studies included
Pulpal blood flow (in perfusion units) at 6 months of molar intrusion Movement vs no movement (force: 100-125 g)		The mean PBF in the intervention groups was0.06 lower (0.36 lower to 0.23 higher)		52 (2 studies)	⊕⊖⊖⊖ very low^1^	Both RCTs and nR PCT studies included
Pulpal blood flow (in perfusion units) at 4 weeks Alignment conventional brackets vs other (self-ligating or aligners)		The mean PBF in the intervention groups was 0.11 higher (0.31 lower to 0.53 higher)		67 (2 studies)	⊕⊖⊖⊖ low^2,3^	Only RCTs included
Aspartate aminotransferase (AST) activity at 7 days (in units/mg of pulp tissue) Movement vs no movement (force: 30-90 g)		The mean AST activity in the intervention groups was 1.65 higher (1.18 lower to 4.47 higher)		76 (2 studies)	⊕⊖⊖⊖ very low^2,3,4,5^	Only nR PCT studies included

*The basis for the assumed risk (e.g. the median control group risk across studies) is provided in footnotes. The corresponding risk (and its 95% confidence interval) is based on the assumed risk in the comparison group and the relative effect of the intervention (and its 95% CI). CI: Confidence interval; PBF: pulpal blood flow; RCT: randomised controlled trial; nR-PCT: non-randomised prospective clinical trial.

GRADE Working Group grades of evidence: High quality: Further research is very unlikely to change our confidence in the estimate of effect. Moderate quality: Further research is likely to have an important impact on our confidence in the estimate of effect and may change the estimate. Low quality: Further research is very likely to have an important impact on our confidence in the estimate of effect and is likely to change the estimate. Very low quality: We are very uncertain about the estimate.

1 two-level downgrade due to RoB 2 one-level downgrade for heterogeneity 3 one-level downgrade due to RoB 4 one-level downgrade due to imprecision 5 no reason for upgrade

## Discussion

### Summary of the Evidence

The findings of the present SR highlight the apparent heterogeneity in the outcomes assessed and their short-term nature. We have attempted to quantify and systematically collate evidence related to potential alterations in the pulp tissue complex when subjected to orthodontic forces. Evidence regarding the most frequently studied outcome in the pool of included studies reveals statistically significant alterations, with decreased pulpal blood flow immediately after and up to three weeks following orthodontic treatment, irrespective of the type of movement, but being more pronounced during moderate (i.e. ≤ 125 grams) intrusive forces. However, these primary alterations of blood microcirculation within the pulp chamber seem to rebound during the course of treatment; the literature reports blood flow values attaining pre-treatment levels 6 months following initiation of orthodontic therapy.^19,46^

Apparently, the swift and immediate effect of tooth movement on the vascular system of the tooth pulp may be attributed to the initial shock and constriction of the blood vessels entering the tissue through the apical foramen or supplementary routes while the tooth is being displaced within its periodontal apparatus. However, this effect has proven to be transient, and teeth under continuous light- to moderate-magnitude force have responded positively and returned to a status of physiological vascular activity within their pulp chambers. A recent study has reported that such findings are very likely to exhibit an age-dependent gradient, with younger patients (under 25 years old) undergoing orthodontic treatment being less prone to develop long-term alterations of the blood flow within the pulp chambers, which usually return to pre-treatment conditions as early as within the first month of treatment.^20^ Prior evidence from histological studies indicates a reduction of pulp cell density with age, followed by a reduction of odontoblasts and morphological changes that may lessen the pulp’s capacity to endure extreme stress.^14^ In this respect, it has been claimed that pulp tissue volume gradually diminishes with age, and this has been correlated with massive apoptotic events of odontoblasts, coinciding with continuous dentin matrix development and dentin deposition changes.^35^ Furthermore, it may be speculated that both type and magnitude of orthodontic tooth movement may pose additional risks if prolonged. Intrusion forces and related mechanics might be regarded as the modalities that could potentially severely adversely affect the vascular status of the pulp, if the blood supply through the apical foramen and pulpal circulation are constantly and extremely impaired.^45^ Based on a recent meta-analysis of moderate to low certainty of evidence, treatment-related factors have been recognised as presenting a minimum effect on root resorption following orthodontic intrusion.^7^

Laser doppler flowmetry (LDF) was employed in the primary studies included here as an accurate means of assessing vascular changes within the pulp. This allowed a non-restricted in-vivo application of the method, eliminated the need to plan subsequent tooth extractions of teeth and avoided further complexity of research design and in-vitro analyses. The latter would compromise the generalisability and extrapolation of the findings to simulated clinical conditions. Furthermore, the transmitted light intensity through dental tissues has been considered adequate to overcome structures such as enamel and dentin, and provide reliable estimates of the vascular pulp activity.^26,32^

Stimuli impacting pulp tissue have also been examined in the context of identifying early signs of inflammatory indicators, by detecting enzymatic activity. Thus, the AST and ALP activity were examined and considerable changes in the expression of these enzymes were documented.^41,42,54^ The gradual increase in AST expression may be considered indicative of increased apoptosis of the osteoblasts as previously reported as well as reduced oxygen levels and lower respiration in the pulp soon after applying orthodontic forces; however, such early alterations might also be indicative of tooth movement-induced proliferation of osteoclasts and mitogenesis.^39,54^ Likewise, a statistically significant decrease in the levels of ALP can probably be attributed to damage and apoptosis of cells involved in the synthesis of ALP, thus rendering the findings consistent with the discussed alterations within the pulp tissue following an orthodontic stimulus and presenting an early odontoblastic degeneration.^41^ Nevertheless, long-term data in this respect are lacking.

Further secretion of substances within the first 24 h of orthodontic force application has also been studied by recent clinical trials.^11,12^ An upsurge in the levels of substance P, CGRP and other related factors has been confirmed irrespective of the force magnitude or type of tooth movement (i.e. intrusion or extrusion). In essence, the levels of these neuropeptides indicate tissue homeostasis and have been considered to play a role in the defense mechanism of the pulp tissue matrix. A rise in their levels has been associated with reparatory conditions, neurogenic inflammation and initiation of mineralised tissue formation; elevated secretion of substance P and CGRP is dependent on the nature and magnitude of the triggering stimuli.^11^ Again, there is no evidence regarding the long-term maintenance of the levels of these peptides. Along the same lines, a single study on maxillary premolars confirmed an increase in c-Fos and matrix metallopeptidase-9 (MMP-9) expression in the dental pulp after being subjected to extreme intrusive force application.^23^ Increased levels of the aforementioned peptides has been shown to be consistent with pulpal blood-vessel dilation, a decrease in cell population accompanied by an increase in fibers, and vacuolisation of odontoblasts.^24,31,37,43^

This SR did not ultimately identify studies confirming loss of pulp vitality after standard orthodontic tooth movement within the timelines assessed. Formation of pulp stones within the pulp chamber were not aetiologically associated with the application of various levels of orthodontic force.^5^ Interestingly, pulp calcification and formation of nodules has been observed in healthy tissues as well, and there is currently no clear aetiologic background in this respect.^13^ According to a previous SR, the presence and formation of pulp stones increased after fixed orthodontic treatment; however, this claim is not substantiated by sound evidence and was based on retrospective or cross-sectional contributing primary studies. Such studies are potentially affected either by confounding factors or a design that cannot support any aetiological link between the effect (orthodontic force) and the outcome (pulp stone formation), as determined by a clearly identified temporal sequence of events.^56^

### Strengths and Limitations

The present study constitutes the first systematic attempt to collate existing evidence on pulp changes induced by orthodontic force application in a quantitative manner. Previous studies in the broader field comprise solely qualitatively synthesised data from various study designs. There are several strengths of the present systematic approach. The protocol was registered a priori, and we also examined only the most rigorous literature: either RCTs or prospective controlled trials. We employed a strict, up-to-date search strategy across 7 databases of published and unpublished literature and included a range of outcomes with no additional search restrictions. Data from both split-mouth and standard parallel-arm study designs were incorporated, after taking into account the decreased within-subject variability according to the related correlation coefficient. The certainty of evidence was assessed, and the data were interpreted based on the quality of evidence in relation to the apparent safety of the interventions studied.

However, the review has some limitations. No meta-analysis incorporated outcomes from more than four studies, with predominantly small trials, so that the precision of the recorded effect estimate is somewhat questionable. All included studies comprise an observation or follow-up period of six months at the most; thus only early effects of orthodontic tooth movement on the pulp tissue complex were evaluated. Although all included studies were prospective, with or without a randomised design, the concerns related to the risk of bias may cast doubt on the validity and interpretation of the present findings.

## Conclusions

Considering all caveats and shortcomings of the available evidence, it seems that early effects of orthodontic force application on pulp tissue matrix do exist. However, they do not pose a significant burden on the tissue, as they are currently reported to be transient. It is essential that further prospective clinical trials focus on the long-term effects of orthodontic treatment on pulpal responses, even after the completion of the orthodontic therapy.

## Appendix 1 Search strategy for all databases

**Table:**

No.	Electronic database	Hits
1.	Medline via Pubmed ((orthodontic tooth movement) OR (orthodontic force) OR (orthodontic movement) OR (tooth movement) OR (teeth movement) OR (tooth biomechanics)) AND ((pulp vitality) OR (pulp necrosis) OR (pulp response) OR (human dental pulp) OR (pulp reaction) OR (pulp* reaction) OR (pulp* response) OR (pulp* vitality) OR (cellular pulp response)) AND ((randomized trial) OR (randomized trial) OR (controlled clinical trial) OR (prospective clinical trial) OR (prospective trial) OR (prospective study) OR (randomized) OR (randomized) OR (prospective))	114
2.	Scopus ((orthodontic tooth movement) OR (orthodontic force) OR (orthodontic movement) OR (tooth movement) OR (teeth movement) OR (tooth biomechanics)) AND ((pulp vitality) OR (pulp necrosis) OR (pulp response) OR (human dental pulp) OR (pulp reaction) OR (pulp* reaction) OR (pulp* response) OR (pulp* vitality) OR (cellular pulp response)) AND ((randomized trial) OR (randomized trial) OR (controlled clinical trial) OR (prospective clinical trial) OR (prospective trial) OR (prospective study) OR (randomized) OR (randomized) OR (prospective))	77
3.	Cochrane Central Register of Controlled Trials (CENTRAL) ((orthodontic tooth movement) OR (orthodontic force) OR (orthodontic movement) OR (tooth movement) OR (teeth movement) OR (tooth biomechanics)) AND ((pulp vitality) OR (pulp necrosis) OR (pulp response) OR (human dental pulp) OR (pulp reaction) OR (pulp* reaction) OR (pulp* response) OR (pulp* vitality) OR (cellular pulp response)) AND ((randomized trial) OR (randomized trial) OR (controlled clinical trial) OR (prospective clinical trial) OR (prospective trial) OR (prospective study) OR (randomized) OR (randomized) OR (prospective))	64
4.	Cochrane Database of Systematic Reviews (CDSR) ((orthodontic tooth movement) OR (orthodontic force) OR (orthodontic movement) OR (tooth movement) OR (teeth movement) OR (tooth biomechanics)) AND ((pulp vitality) OR (pulp necrosis) OR (pulp response) OR (human dental pulp) OR (pulp reaction) OR (pulp* reaction) OR (pulp* response) OR (pulp* vitality) OR (cellular pulp response)) AND ((randomized trial) OR (randomized trial) OR (controlled clinical trial) OR (prospective clinical trial) OR (prospective trial) OR (prospective study) OR (randomized) OR (randomized) OR (prospective))	2
5.	LILACS (Virtual Health Library: https://lilacs.bvsalud.org/en/) ((orthodontic tooth movement) OR (orthodontic force) OR (orthodontic movement) OR (tooth movement) OR (teeth movement) OR (tooth biomechanics)) AND ((pulp vitality) OR (pulp necrosis) OR (pulp response) OR (human dental pulp) OR (pulp reaction) OR (pulp* reaction) OR (pulp* response) OR (pulp* vitality) OR (cellular pulp response)) AND ((randomized trial) OR (randomized trial) OR (controlled clinical trial) OR (prospective clinical trial) OR (prospective trial) OR (prospective study) OR (randomized) OR (randomized) OR (prospective))	74
6.	ClinicalTrials.gov (www.clinicaltrials.gov) (orthodontic) AND (pulp)	19
7.	National Research Register (ISRCTN: www.controlled-trials.com) (orthodontic) AND (pulp)	7
